# Measurement properties of EQ-5D-3L and EQ-5D-5L in recording self-reported health status in older patients with substantial multimorbidity and polypharmacy

**DOI:** 10.1186/s12955-020-01564-0

**Published:** 2020-09-29

**Authors:** Arjun Bhadhuri, Paul Kind, Paola Salari, Katharina Tabea Jungo, Benoît Boland, Stephen Byrne, Stefanie Hossmann, Olivia Dalleur, Wilma Knol, Elisavet Moutzouri, Denis O’Mahony, Kevin D. Murphy, Linda Wisselink, Nicolas Rodondi, Matthias Schwenkglenks

**Affiliations:** 1 Institute of Pharmaceutical Medicine (ECPM), University of Basel, Basel, Switzerland; 2 Academic Unit for Health Economics, Institute for Health Sciences, University of Leeds, Leeds, UK; 3 Institute of Primary Health Care (BIHAM), University of Bern, Bern, Switzerland; 4 Institut de Recherche Santé et Société, Université Catholique de Louvain, Brussels, Belgium; 5 Pharmaceutical Care Research Group, School of Pharmacy, University College Cork, Cork, Ireland; 6 Department of Geriatric Medicine and Expertise Centre Pharmacotherapy in Old Persons, University Medical Center Utrecht, Utrecht University, Utrecht, The Netherlands; 7 Department of General Internal Medicine, Inselspital, Bern University Hospital, University of Bern, Bern, Switzerland; 8 CTU Bern, University of Bern, Bern, Switzerland; 9 Department of Medicine (Geriatrics), School of Medicine, University College Cork, Cork, Ireland

## Abstract

**Background:**

The EQ-5D-3L and EQ-5D-5L are two generic health-related quality of life measures, which may be used in clinical and health economic research. They measure impairment in 5 aspects of health: mobility, self-care, usual activities, pain/discomfort and anxiety/depression. The aim of this study was to assess the performance of the EQ-5D-3L and EQ-5D-5L in measuring the self-reported health status of older patients with substantial multimorbidity and associated polypharmacy.

**Methods:**

Between 2017 and 2019, we administered EQ-5D-3L and EQ-5D-5L to a subset of patients participating in the OPERAM trial at 6 months and 12 months after enrolment. The OPERAM trial is a two-arm multinational cluster randomised controlled trial of structured medication review assisted by a software-based decision support system versus usual pharmaceutical care, for older people (aged ≥ 70 years) with multimorbidity and polypharmacy. In the psychometric analyses, we only included participants who completed the measures in full at 6 and 12 months. We assessed whether responses to the measures were consistent by assessing the proportion of EQ-5D-5L responses, which were 2 or more levels away from that person’s EQ-5D-3L response. We also compared the measures in terms of informativity, and discriminant validity and responsiveness relative to the Barthel Index, which measures independence in activities of daily living.

**Results:**

224 patients (mean age of 77 years; 56% male) were included in the psychometric analyses. Ceiling effects reported with the EQ-5D-5L (22%) were lower than with the EQ-5D-3L (29%). For the mobility item, the EQ-5D-5L demonstrated better informativity (Shannon’s evenness index score of 0.86) than the EQ-5D-3L (Shannon’s evenness index score of 0.69). Both the 3L and 5L versions of EQ-5D demonstrated good performance in terms of discriminant validity, i.e. (out of all items of the EQ-5D-3L and EQ-5D-5L, the pain/discomfort and anxiety/depression items had the weakest correlation with the Barthel Index. Both the 3L and 5L versions of EQ-5D demonstrated good responsiveness to changes in the Barthel Index.

**Conclusion:**

Both EQ-5D-3L and EQ-5D-5L demonstrated validity and responsiveness when administered to older adults with substantial multimorbidity and polypharmacy who were able to complete the measures.

## Introduction

Economic evaluations in health care involve the comparison of the costs and the benefits of different health technologies [1]. Cost-effectiveness analysis is a widely accepted form of economic evaluation. Cost-utility analysis (CUA) is a specific form of cost-effectiveness analysis in which the benefits of health technologies are measured in terms of quality adjusted life years (QALYs) [1]. The QALY is a composite measure of both quantity and quality of life.

EQ-5D is a generic measure of health-related quality of life (HrQoL) which can be used in clinical and economic studies, and is the recommended measure in National Institute of Health and Care Excellence (NICE) guidelines for calculating QALYs in cost-utility analysis in England and Wales [2]. EQ-5D consists of 5 dimensions of health i.e. mobility, self-care, usual activities, pain/discomfort and anxiety/depression [3]. It also includes a visual analogue scale (EQ-VAS), which asks participants to rate their overall health on a scale from 0 to 100. In the original of the EQ-5D (EQ-5D-3L), each dimension of health included 3 answer options (levels) to measure whether participants were experiencing no problems, some/moderate problems or severe/extreme problems [3]. However, there were concerns that the use of only 3-levels resulted in these levels being too broad so that the EQ-5D-3L measure offered only limited information on the degree to which respondents’ health was impaired, and was also less sensitive to changes in respondents’ health status over time. As a result, a 5-level version of the EQ-5D (EQ-5D-5L) was subsequently developed and introduced in 2009 to address these concerns by providing two additional levels for each dimension to enable a more nuanced profile of an individual’s health status to be elicited. In the EQ-5D-5L, each dimension of health includes 5 levels to measure whether participants are experiencing no problems, slight problems, moderate problems, severe problems, or extreme/unable problems [4]. Henceforth in this article, we refer to the EQ-5D-3L and EQ-5D-5L instruments as 3L and 5L respectively.

For the purposes of economic evaluation, EQ-5D responses can be converted into a single index summary score based on questionnaire responses to the 5 dimensions of health by using a valuation algorithm based on the social preferences of the general population. Such evaluation algorithms are country-specific and are currently available for a number of countries.

The measurement properties of any HrQoL instrument, such as distributional properties, consistency, reliability and validity, should be evaluated in order to assess its appropriateness for use in a specific patient population [5]. A measurement instrument may exhibit good distributional properties if the presence of ceiling and floor effects are low (so that responses are not concentrated within the highest and/or lowest levels of an instrument). Both 3L and 5L should demonstrate consistency with each other if participants’ responses to the 5L matched with the corresponding levels of the 3L when both measures were administered at the same time point [6]. Reliability analysis assesses the ability of an instrument to provide reproducible measurements, whereas validity analysis involves assessing the extent to which an instrument measures what it purports to measure [7]. Convergent (and discriminant) validity and responsiveness are two types of validity analysis. An instrument exhibits convergent validity if it is highly correlated with a related instrument, whereas an instrument exhibits discriminant validity if it has a comparatively low correlation with an unrelated instrument [5].

Responsiveness may be described as ‘longitudinal validity’, and assesses the degree to which an instrument is able to respond to a meaningful or clinically important external change over time [5, 8]. Given the most common function of the EQ-5D is to detect changes in HrQoL over time in clinical trials, it is particularly important to evaluate the responsiveness of EQ-5D. An anchor-based analysis may be performed to assess responsiveness. The objective of an anchor-based analysis is to assess whether scores on the measure of interest (i.e. 3L or 5L) change in the expected direction when compared with changes in the scores of a related construct or measure (the ‘anchor’ measure) [9, 10]. For an anchor-based responsiveness analysis to be undertaken, it is necessary that the anchor measure is responsive in the study population.

We are aware of two previous studies which have compared the 3L and 5L versions of EQ-5D for people of any age with multimorbidity (defined in these studies as ≥ 2 chronic conditions) [11, 12]. Our study is the first we are aware of, to examine the responsiveness of the 3L or 5L in a population with substantial multimorbidity (our sample presented with a mean of 11.5 chronic conditions upon entry into our study) and polypharmacy (defined for this study, as 5 or more different regular drugs for more than 30 days). The definition of multimorbidity used in this study was based on the inclusion criteria for the underlying clinical trial that provided the data basis for the present study (presence of ≥ 3 concurrent chronic conditions), and is stricter than the definition of multimorbidity which is typically used in the clinical field (presence of ≥ 2 concurrent chronic conditions) [13, 14]. Studying the measurement properties of the 3L and 5L in this population is of significant interest, because this population is increasing in prevalence over time [15]. Our study is also the first head-to-head study we are aware of (i.e. the same individual completing both the 3L and 5L) that has been undertaken for this population. In terms of studies comparing the measurement properties of 3L and 5L versions of EQ-5D, many studies have been carried out across other populations [16]. Most of these studies showed that the 5L is highly consistent with 3L responses, as well as offering a better level of performance in terms of reduced ceiling effects and better informativity compared to the 3L [16–18]. Ceiling effects occur when a high proportion of subjects have maximum scores on the measurement of interest. A smaller number of studies, which have applied modern test theory through Rasch analysis, have also indicated improved performance of the 5L compared to the 3L in terms of demonstrating greater sensitivity [19, 20]. Furthermore, we are aware of only six studies comparing the responsiveness of the 5L and 3L. Of these, three studies found that the 5L was more responsive than the 3L [21–23], two found that the measures exhibited similar responsiveness [24, 25], and one study of 112 stroke patients indicated that the 3L was more responsive than the 5L [26].

The main objectives of this study were to:

a Assess discriminant validity, informativity and responsiveness of the 3L and 5L versions of EQ-5D in an older adult population with substantial multimorbidity, and polypharmacy.
b Assess consistency of the 3L and 5L, in an older adult population with substantial multimorbidity, and polypharmacy. Consistency involves assessing the extent to which responses based on 3L correspond to those based on 5L.

## Methods

### Data collection

The OPERAM clinical trial is a two-arm, cluster-randomised controlled trial of a structured medication review assisted by a software-based decision support system versus usual care, funded by the European Union Horizon 2020 programme (trial identifier: NCT02986425) [14]. The trial was conducted in four centres in Belgium, Ireland, Netherlands, and Switzerland with a follow-up period of 12 months. The trial participants were 2,008 people aged 70 years or above with both multimorbidity (experiencing 3 or more chronic conditions concurrently) and polypharmacy (5 or more different regular drugs) [14]. The trial intervention was based on the so-called ‘Systematic Tool to Reduce Inappropriate Prescribing’ (STRIP) assistant, which is deployed using a clinical decision support system [27]. STRIP is a structured method for performing customised medication reviews and to detect potentially inappropriate prescribing [14], based on STOPP/START version 2 criteria for potentially inappropriate medications (STOPP) and potential prescribing omissions (START) [28]. Baseline characteristics of the OPERAM trial participants included a mean age of 80 years, 55% being male, 24% being university educated, and presenting with a mean of 11.0 comorbidities at baseline. The chronic comorbid conditions most commonly reported by OPERAM trial participants at baseline were hypertension (n = 1309; 65%), hypercholesterolemia (n = 725; 36%) and atrial fibrillation (n = 724; 36%). The OPERAM trial had broad eligibility criteria to improve representativeness for the population of interest, and external validity.

During the 6 month and 12 month participant telephone interviews, which took place between 2017 and 2019, all trial participants were asked to verbally complete questionnaires read out to them by the trial primary researcher, to elicit a range of trial outcomes (including the Barthel Index, EQ-VAS and EQ-5D-5L). The EQ-5D-5L was included in the OPERAM trial as part of a pre-planned, within-trial health economic analysis, but also to assess HRQoL clinically [14] (primarily by using the EQ-VAS). In addition, after initial trial recruitment had been completed, the 3L questionnaire was administered in the same way to a subset of participants of the OPERAM trial at the 6 month and 12 month follow-up time points. We chose these time points to collect EQ-5D data for assessment of responsiveness, as we judged that a 6 month interval was sufficient time for clinically important changes in patient’s health to occur. We did not use a longer time period, as it would have exacerbated the generation of missing data due to the substantial mortality rate in the target population. Based on the standard operating procedure for the administration of trial questionnaires, the 3L was administered at the end of the telephone interview. For patients with potential difficulty in concentrating, the 5L was the first questionnaire administered, followed in sequence by (1) the Morisky Medication Adherence Scale (MMAS-8), (2) the Barthel Index and (3) Beliefs about Medicines Questionnaire (BMQ).

In the course of the 6 month follow-up interviews, patients were consecutively added to the present study until a maximum of 75 participants was reached for each country [implying a planned maximum of 300 participants in total; 300 being comparable to the sample size of other responsiveness studies comparing 3L and 5L [16]]. In this study nested within a multinational clinical trial, we used the combined sample across all countries to ensure sufficient statistical power. However, for the responsiveness analyses, we also carried out subgroup analyses at the country level to check for potential differences in the responsiveness of the instruments between countries. Questionnaires were completed by patients or proxies on behalf of the patient, usually a family member or other responsible individual [i.e. nursing home employee (if applicable) or the patient’s GP [14]] if the patient presented with cognitive impairment or was otherwise unable to respond. However, our present analysis was restricted to participants who self-completed the EQ-5D measures at 6 and 12 months. It was considered necessary to remove participants for whom a proxy EQ-5D report was obtained, as they were shown to have a markedly different health profile compared to participants who self-completed all EQ-5Ds, reflected by them having statistically significant lower 6 month Barthel Index score (i.e. greater impairments in activities of daily living; *p* < 0.001). Another reason for removing proxy EQ-5D responses was that these can be divergent from self-completed EQ-5D responses [29]. The inclusion of the proxy responses might have led to a situation where observed differences between 3 and 5L could partially be driven by the proxy responses, with no sufficient possibility to distinguish this. Therefore, we regarded it as more appropriate to focus on the responses directly provided by patients. Ethical approval for the study was obtained at the four OPERAM clinical sites.

### Calculation of EQ-5D scores

To be consistent and because no equivalent value set exists for Switzerland, we used German EQ-5D value sets for all analyses. The German time trade-off value set was used to calculate 3L scores (utilities) [30], and the German cross-walk algorithm was used to calculate 5L scores [31]. The German crosswalk algorithm maps 5L responses onto the German 3L value set to calculate 5L scores.

### Statistical analysis

All psychometric analyses were restricted to participants who self-completed all items of the 3L and 5L instruments at 6 months and 12 months.

### Descriptive statistics

Descriptive statistics were calculated for the study sample, including participant characteristics and the distribution of participants across all of the levels and dimensions of the 3L and 5L at baseline (6 month responses) and follow-up (12 month responses) [26]. Volume and patterns of missing data for the 3L and the 5L were assessed. We also calculated correlation coefficients between the 3L and 5L index scores, between the 3L and VAS, and between the 5L and VAS. A very high correlation between the 3L and 5L index scores might indicate the instruments produce similar results and imply that they could be used interchangeably [32].

### Consistency and redistribution properties

The consistency of the EQ-5D at 6 months (i.e. first measurement time point) was evaluated by cross-tabulating within-participant 3L and 5L responses. An inconsistent response was defined as a 5L response that was two or more levels away from the corresponding 3L response [6]. For example, an inconsistent response would be established for a participant who reported level 1 (no problems) using 3L but reported level 3 (some problems) or worse for the same dimension using 5L. An exception to this rule was made for the mobility item. Here, we considered responses from participants who reported with the 3L some problems in walking about, and also reported with the 5L being unable to walk about, to *not* be inconsistent. This is because the 3L mobility item is categorised into a person having “no problems in walking about”, “some problems in walking about” and being “confined to bed”. Patients who report being unable to walk about with the 5L, may not necessarily be confined to bed and may therefore instead logically report having “some problems in walking about” with the 3L.

The proportions of inconsistent responses for each of the dimensions were computed. For consistent responses, the redistribution properties of the 5L were also assessed in the cross-tabulation. For example, we were able to assess the redistribution of participants who reported ‘some problems’ for a 3L dimension, across the ‘some problems’, ‘moderate problems’ and ‘severe problems’ levels of the corresponding 5L dimension.

### Ceiling effects

The proportion of participants who reported ‘no problems’ for each dimension of the 3L and the 5L was assessed. We also examined the proportion of participants who reported no problems for *all* dimensions of 3L and 5L (i.e. index scores of 1). McNemar’s test was used to test whether there were statistically significant differences in ceiling effects between the measures for each dimension [33]. A previous study of the general German population found that approximately 39% of respondents aged 70–79 years and 7.6% of respondents aged 80+ years reported ‘no problems’ for all 5 items of the EQ-5D-5L [34].

### Discriminant validity

The discriminant validity of the EQ-5D-3L and 5L was assessed by computing Spearman’s rho between each of the EQ-5D items, and the Barthel Index at 6 months [33]. The Barthel Index is a measure of individual performance in activities of daily living (ADLs) widely used in the field of rehabilitation, consisting of 10 items. Barthel Index scores range from 0 (indicating ‘total’ dependency in ADLs) to 100 (indicating no dependency in ADLs) [35]. Spearman’s rho effect sizes of between 0.20 and 0.35 were considered weak, between 0.35 and 0.50 moderate and > 0.50 strong [33]. We assessed discriminant validity for the 3L and the 5L by testing the hypothesis that Spearman’s rho for the EQ-5D anxiety/depression or pain/discomfort items with the Barthel Index would be lower than for the other EQ-5D items. This is because the other EQ-5D items (mobility, self-care, usual activities) measure functioning, thereby being expected to correlate better with the Barthel Index which measures ADL-related functioning [36].

### Responsiveness

The responsiveness of the EQ-5D-3L and 5L measures to changes in the Barthel Index and the EQ-VAS over time (i.e. between 6 and 12 months) was assessed by using an anchor-based analysis [8]. The Barthel Index and EQ-VAS were also secondary outcome measures in the OPERAM trial [14], due to their perceived responsiveness in the OPERAM population. The EQ-VAS is a visual analogue scale measure of a person’s self-assessed health with status ranging from 0 to 100 [37]. The ‘anchor’ measures (Barthel Index and EQ-VAS) were each sub-divided into three categories to reflect whether (1) the participant’s score for the anchor measure improved clinically, (2) did not change in a clinically important way, or (3) clinically worsened between 6 and 12 months. The threshold for a clinically important change was determined using a literature-based minimal clinically important difference (MCID) estimate of 8 points for the EQ-VAS [37], and any change in the total score of the Barthel Index can be considered clinically important [38]. Standardised effect sizes (Cohen’s D) were calculated for changes in EQ-5D scores between 6 and 12 months. Cohen’s D effect sizes of between 0.2 and 0.5 were considered small, 0.5 and 0.8 moderate and > 0.8 large [39]. A high degree of responsiveness of the EQ-5D-3L/5L measures would be indicated through their demonstrated ability to detect change in the anchor measures, i.e. positive effect sizes (moderate or large) for the EQ-5D when there is an improvement in the anchor measure and negative effect sizes when there is a worsening in the anchor measure. In our study, both the EQ-5D-3L and EQ-5D-5L were administered in full, including their VAS parts that are introduced slightly differently. We assessed responsiveness of the EQ-5D-3L to change in the 3L-VAS measure, and responsiveness of the EQ-5D-5L to change in the 5L-VAS measure, as we observed differences between 3L-VAS responses and 5L-VAS responses elicited at the 6 month time point, in 15 out of the 224 participants in our sample (although we observed no differences between 3L-VAS responses and 5L-VAS responses at the 6 month time point in 209 out of the 224 participants, suggesting that broadly, the VAS can still be considered a common anchor measure for our analysis). The 3L-VAS and the 5L-VAS measures are for all essential purposes, identical measures.

### Informativity

Informativity of the EQ-5D-3L and the 5L measures were assessed at 6 months using the Shannon index (H′) and the Shannon evenness index (J′) [40]. H′ was calculated for each dimension of the 5L using the formula: H′ = − (proportion_none*log2(proportion_none) + proportion_some*log2(proportion_some) + proportion_moderate*log2(proportion_moderate) + proportion_severe*log2(proportion_severe) + proportion_extreme/unable*log2(proportion_extreme/unable), and similarly calculated for the 3L dimensions [21]. Higher H′ values indicate that responses to the dimension are more evenly spread across the different categories of the dimension, and consequently suggest greater informativity. The formula for the Shannon evenness index is: J′ = H′/H′max. The value of H’max for the 3L is log2(3) = 1.58 and for the 5L is log2(5) = 2.32. Unlike H′ values, J′ values lie on a common 0 to 1 scale allowing for direct comparison of results from the 3L with the 5L.

## Results

### Descriptive statistics

At the 6 months follow-up in the OPERAM study, 256 (83%) of patients reported the EQ-5D measures themselves, 45 (15%) had the EQ-5D measures reported by proxy by their next of kin, and 8 (2%) had the EQ-5D measures reported by proxy by some other individual (unspecified). Of the 256 participants, 224 participants also self-reported EQ-5D measures at 12 months, and with full completion of all 3L and 5L items at 6 and 12 months. This sample of 224 participants was used for all analyses and included participants who reported inconsistent responses. Age, gender, education level and comorbidity characteristics of the sample analysed for this study, were broadly similar to the characteristics of the overall OPERAM trial population (described in the methods section).

Summary statistics are provided in Table 1, showing that 56% of participants were male, 28% were university educated, the highest level of education was completed high school for 46% of participants, 26% of participants did not complete high school and 5% had spent some time in the 6 months before the trial started living in a nursing home. The average participant was experiencing a median of 10 coexistent chronic conditions upon entering the OPERAM trial. A small index score reduction of 0.01 (rounded) was observed between 6 and 12 months for both the 3L and 5L.

**Table 1:** Summary statistics (n = 224)

Variable	n	Mean	SD
Age (years)	224	77.5	5.35
Number of coexistent chronic conditions^a^	224	11.5 (median = 10)	6.01
Number of medications^a^	224	9.3	3.34
Barthel Index 6 months	224	95.2	8.81
EQ-5D-3L index score 6 months	224	0.83	0.21
EQ-5D-3L index score 12 months	224	0.82	0.22
EQ-5D-5L index score 6 months	224	0.81	0.19
EQ-5D-5L index score 12 months	224	0.80	0.21
EQ-VAS score 6 months	224	69.9	15.71

Variable	n (%)
Gender (male)	126 (56.2%)
Highest level of education—university	62 (27.6%)
Highest level of education—high school	102 (45.5%)
Highest level of education—less than high school	59 (26.3%)
Highest level of education—not applicable/reported	1 (0.4%)
Spent some time in the 6 months before trial in nursing home	10 (4.4%)
Country of residence—Switzerland	55 (24.5%)
Country of residence—Ireland	48 (21.4%)
Country of residence—Belgium	58 (25.8%)
Country of residence—Netherlands	63 (28.1%)

*SD* standard deviation

^a^At OPERAM trial baseline

In this sample at 6 months, 41 unique health states were represented using the EQ-5D-3L, and 99 states using the EQ-5D-5L. Spearman’s rho at 6 months between the 3L and 5L index scores was 0.88 (95% CI: 0.84 to 0.90), between the 3L index scores and 3L-VAS was 0.41 (95% CI: 0.30 to 0.52), and between the 5L index score and 5L-VAS was 0.44 (95% CI: 0.32 to 0.54).

Missing data was similar between both instruments (see footnotes of Appendix Tables 7 and 8).

With both the 3L and 5L, it was observed that there was a small reduction between the 6 and 12 month time points in the rate of participants reporting "no problems" in their ability to undertake usual activities [from 73 to 68% with the 3L (Appendix Table 7), and from 64 to 61% with the 5L (Appendix Table 8)]. There were no statistically significant changes at the 5% level in responses between 6 and 12 months, for any of the 3L and 5L dimensions (Appendix Tables 7 and 8). Whilst the pattern of change as indicated by 3L and 5L between the two time points is broadly similar, there were important differences. Notably, it was observed that for mobility and anxiety/depression, the direction of change was different between 3 and 5L (positive for 3L and negative for 5L for both items; see Appendix Table 9).

### Consistency

We assessed presence of inconsistent responses between 3 and 5L, i.e. 5L responses that differed by ≥ 2 levels with the same person’s 3L response (highlighted in Appendix Table 10). There were 28 (3%) inconsistent responses between the 3L and 5L reported across items (7 (3%) inconsistent responses for the mobility item, 4 (2%) for the self-care item, 7 (3%) for the usual activities item, 4 (2%) for the pain/discomfort item and 4 (2%) for the anxiety/depression item). The 28 inconsistent responses were elicited from 26 participants in total.

### Ceiling effects

A high presence of ceiling effect was observed for the self-care item for both instruments (84% of participants reported "no problems" for self-care with the EQ-5D-3L and 83% with the EQ-5D-5L). There was a substantially higher degree of ceiling effect with the EQ-5D-3L index score (29%) than with the EQ-5D-5L index score (22%), which was a statistically significant difference (*p* < 0.001) (Table 2).

**Table 2:** Percentage of patients with a ceiling effect for each dimension of the completed EQ-5D-3L and EQ-5D-5L instruments and for the overall measures at 6 months (n = 224)

EQ-5D dimension	EQ-5D-3L (%)	EQ-5D-5L (%)	*p* values*
Mobility	50.8	39.2	** < ***0.001*
Self-care	84.3	83.4	0.48
Usual activities	72.7	63.8	**< ***0.001*
Pain/discomfort	50.4	44.6	*0.002*
Anxiety/depression	83.4	79.0	*0.003*
Index score	29.4	22.3	**< ***0.001*

*Probability values presented in italic indicate significant decreases in ceiling effects from using the EQ-5D-5L measure compared with the EQ-5D-3L measure (calculated using McNemar’s test)

For comparison, 4 participants (2%) reported a VAS score of 100 at 6 months (indicating they have the ‘best health they can imagine’). All 4 of these participants also reported full health with both the 3L and 5L at 6 months. 81 participants (36%) reported a Barthel Index score of 100 at 6 months (indicating they have no dependency in ADLs). Of these, 58 participants reported full health with the 3L, and 44 participants reported full health with the 5L at 6 months.

### Validity

For discriminant validity, we assessed the correlation between the EQ-5D items and Barthel Index (Table 3). There were no statistically significant differences at the 5% level between the 3L and 5L items, in terms of how correlated they were with the Barthel Index (absence of statistically significant differences was demonstrated from all 95% confidence intervals for the 3L items overlapping with the 95% confidence intervals for the corresponding 5L items). Although the difference was not statistically significant, it is observed that the negative correlation between the mobility domain and the Barthel index was larger in magnitude for the 5L. We found that out of all items of the 3L and 5L, the pain/discomfort and anxiety/depression items had the weakest correlation with the Barthel Index.

**Table 3:** Correlation coefficients of dimensions of the EQ-5D measures with the Barthel Index (n = 224)

Dimension	R_S_ between 3L and Barthel Index^a,b^	95% CI	R_S_ between 5L and Barthel Index^a,b^	95% CI
Mobility	− 0.37	− 0.48 to − 0.25	− 0.42	− 0.52 to − 0.30
Selfcare	− 0.57	− 0.65 to − 0.48	− 0.58	− 0.66 to − 0.48
Usual activities	− 0.45	− 0.55 to − 0.34	− 0.42	− 0.52 to − 0.31
Pain/discomfort	− 0.15	− 0.27 to − 0.02	− 0.16	− 0.28 to − 0.03
Anxiety/depression	− 0.14	− 0.27 to − 0.01	− 0.13	− 0.26 to − 0.003

*CI *confidence interval

^a^Spearman’s rho effect sizes of between 0.20 and 0.35 are considered weak, between 0.35 and 0.50 moderate, > 0.50 strong

^b^Higher EQ-5D item score indicates worse health problems; higher EQ-5D index score indicates better health

### Responsiveness

142 participants (64%) reported no change in their total Barthel Index score between 6 and 12 months. Responsiveness analysis demonstrated both EQ-5D measures were responsive to changes in the Barthel Index from 6 to 12 months (Table 4). Evidence of responsiveness of both measures to changes in the Barthel Index, was demonstrated both in the overall sample (Table 4), as well as in each of the country-specific subgroups (Appendix Tables 11, 12, 13, 14). Furthermore, compared to each other, both 3L and 5L measures were similar in their responsiveness to changes in the Barthel Index. For both the 3L and 5L, Cohen’s D effect sizes changed from a moderate positive effect when the Barthel Index improved to a small negative effect when the Barthel Index worsened.

**Table 4:** Assessment of responsiveness of the EQ-5D-3L and the EQ-5D-5L measures to changes in the Barthel Index (n = 224)

	EQ-5D-3L at 6 months (mean)	EQ-5D-3L at 12 months (mean)	Difference between 12 and 6 months EQ-5D-3L (95% CI)	Effect size (Cohen’s D)^a^	n	% reporting 3L score increase
Barthel Index						
Improved	0.68	0.83	0.16 (0.07 to 0.25)	0.66**	31	55
No change	0.89	0.87	− 0.02 (− 0.05 to 0.02)	− 0.09	142	25
Worsened	0.74	0.66	− 0.08 (− 0.16 to 0.00)	− 0.30*	50	28

	EQ-5D-5L at 6 months (mean)	EQ-5D-5L at 12 months (mean)	Difference between 12 and 6 months EQ-5D-5L (95% CI)	Effect size (Cohen’s D)	n	% reporting 5L score increase
Barthel Index						
Improved	0.68	0.81	0.13 (0.07 to 0.20)	0.66***	31	59
No change	0.87	0.86	− 0.01 (− 0.04 to 0.02)	− 0.05	142	32
Worsened	0.73	0.65	− 0.09 (− 0.14 to − 0.03)	− 0.36**	50	32

^a^Cohen’s D effect sizes of between 0.2 and 0.5 are considered small, 0.5 and 0.8 moderate and > 0.8 large

*p < 0.05; **p < 0.01; ***p < 0.001

Both the 3L and 5L demonstrated some degree of responsiveness to changes in the VAS from 6 to 12 months (Table 5). Compared with each other, both 3L and 5L measures demonstrated similar responsiveness to changes in the VAS. There was a small positive improvement in 3L and 5L scores as the patient’s VAS scores improved. This improvement in the overall sample appeared to be driven by improvements in 3L and 5L scores in the Netherlands and Ireland (Appendix Tables 17, 18). However, there was no statistically significant change in in 3L and 5L scores for the patients whose VAS scores worsened from 6 to 12 months.

**Table 5:** Assessment of responsiveness of the EQ-5D-3L and the EQ-5D-5L measures to changes in the VAS (n = 224)

	EQ-5D-3L at 6 months (mean)	EQ-5D-3L at 12 months (mean)	Difference between 12 and 6 months EQ-5D-3L (95% CI)	Effect size (Cohen’s D)	n	% reporting 3L score increase
3L-VAS						
Improved	0.80	0.88	0.08 (0.02 to 0.14)	0.40*	52	44
No MCID	0.85	0.81	− 0.04 (− 0.10 to 0.01)	− 0.19	101	30
Worsened	0.81	0.79	− 0.02 (− 0.06 to 0.02)	− 0.07	71	21

	EQ-5D-5L at 6 months (mean)	EQ-5D-5L at 12 months (mean)	Difference between 12 and 6 months EQ-5D-5L (95% CI)	Effect size (Cohen’s D)	N	% reporting 5L score increase
5L-VAS						
Improved	0.78	0.84	0.06 (0.01 to 0.10)	0.31*	55	45
No MCID	0.83	0.81	− 0.03 (− 0.07 to 0.02)	− 0.13	98	36
Worsened	0.80	0.78	− 0.02 (− 0.06 to 0.02)	− 0.11	71	28

*MCID *minimal clinically important change

^a^Cohen’s D effect sizes of between 0.2 and 0.5 are considered small, 0.5 and 0.8 moderate and > 0.8 large

*p < 0.05; **p < 0.01

### Informativity

Shannon’s evenness indices indicated that the 3L and 5L were informative for mobility, usual activities and pain/discomfort dimensions, although less informative for self-care and anxiety/depression dimensions (Table 6). The EQ-5D-3L was slightly more informative with respect to self-care (EQ-5D-3L J′ = 0.48; EQ-5D-5L J′ = 0.42), and the EQ-5D-5L was substantially more informative with respect to mobility (EQ-5D-3L J′ = 0.69; EQ-5D-5L J′ = 0.86).

**Table 6:** Shannon’s index (H′) and Shannon’s evenness index (J′) values for EQ-5D-3L and EQ-5D-5L measures at 6 months

Dimension	3L			5L		
	No. of patients	H′	J′	No. of patients	H′	J′
Mobility	224	1.09	0.69	224	1.99	0.86
Self-care	224	0.75	0.48	224	0.96	0.42
Usual activities	224	0.94	0.59	224	1.51	0.65
Pain/discomfort	224	1.27	0.80	224	1.85	0.80
Anxiety/depression	224	0.73	0.46	224	1.05	0.45

## Discussion

In this study, we investigated the measurement properties of the EQ-5D-3L and EQ-5D-5L in older adults with substantial multimorbidity. From our analyses, we found a low proportion of inconsistent responses between the EQ-5D-3L and EQ-5D-5L, which was also found in the majority of previous studies comparing the 3L and 5L [16]. This indicates 5L responses distribute logically with the 3L responses. The EQ-5D-3L represented 41 unique health states out of a possible 243 states (17%), and the EQ-5D-5L represented 99 unique health states out of a possible 3,125 states (3%). This shows that more of the descriptive space of the 3L is used. Both the EQ-5D-3L and EQ-5D-5L exhibited discriminant validity with the Barthel Index; which was also found in a previous study [36]. Missing data occurrence at 12 months was also similar between the two measures. Almost all missing data resulted from participants not being available at 12 months to provide necessary information for secondary outcome measures of the main OPERAM trial through telephone interview (e.g. due to trial drop-out), and should not be considered reflective of the performance of the EQ-5D measures themselves.

We observed high rates of ‘no problems’ with 3L and 5L self-care and anxiety/depression items, which could suggest that the EQ-5D description of levels excludes the type of self-care or anxiety/depression problems encountered by the patient population studied. Alternatively, it may be the case that patients genuinely do not have such problems, or that care settings are working well to enable self-care.

Consistent with most other studies [16, 41, 42] including an assessment of the subgroup of multimorbid patients in a study by Thompson et al. [11], in our sample we observed a reduction in ceiling effects from using the EQ-5D-5L (22%) compared to the EQ-5D-3L (29%). The EQ-5D-5L therefore appears to better capture variability in health status among those who have a high level of health, compared with the EQ-5D-3L. Also consistent with all the studies identified in a systematic review by Buchholz et al. in 2018 [16] was our finding of an overall improvement in informativity from using the 5L compared to the 3L. This was the consequence of a substantially higher Shannon evenness index score for the mobility item of the 5L compared with the 3L; which was also observed in a study of multimorbid adults by Thompson et al. [11]. However, informativity in our study was higher on the 3L than with the 5L for self-care.

We observed similar responsiveness to change over time for the EQ-5D-3L and EQ-5D-5L. Several studies evaluating responsiveness have reported an improvement in responsiveness from using the EQ-5D-5L compared with the EQ-5D-3L [21–23], but other studies have reported either no difference in responsiveness [24, 25] or a reduced responsiveness from using the EQ-5D-5L compared with the EQ-5D-3L [26]. Given the mixed findings across these responsiveness studies, there is currently no clear evidence that using the 5L instead of the 3L to collect utility data for economic evaluations, will lead to systemically different incremental QALY estimates. This contrasts with the notion by Hernandez-Alava et al. (2018), that using the 5L instead of the 3L will lead to systemically lower estimates of incremental QALYs [43]. In our study, both the 3L and 5L were more responsive to the Barthel Index than they were to the VAS. This may be because the VAS measures a broader underlying construct of health, whereas the Barthel Index is a disability-specific measure. Feng et al. also previously observed a weak correlation between EQ-VAS change scores with 5L change scores [44].

This is the first study to investigate measurement properties of the EQ-5D-3L and EQ-5D-5L in older adults with substantial multimorbidity, through a head-to-head comparison. The 5L and 3L were not administered directly after each other, which probably reduced the possibility of a patient’s 3L response being directly influenced by the 5L response immediately beforehand. Further separation was not possible given the set-up of the OPERAM trial. We were able to carry out a robust assessment of responsiveness through analysis of a sample of 224 participants who we assessed over a 6-month follow-up period. We assessed responsiveness within a clinical trial, and observed for our sub-sample, only a very small reduction in 3L and 5L scores between 6 and 12 months. The findings of our study may to an extent be relevant to other clinical trials during which small changes in health are occurring (particularly in trials with a similar population to our own), and inform the decision of whether to select the 3L or 5L in such trials. A limitation of our study is that we only investigated responsiveness of the instruments to changes in the Barthel Index and the EQ-VAS. Investigation of responsiveness of the instruments to other variables predicted to correlate with HRQoL in older multimorbid patients would have been desirable but these were not available. In our analyses, we assessed responsiveness of the EQ-5D-3L to change in the 3L-VAS measure, and responsiveness of the EQ-5D-5L to change in the 5L-VAS measure. We did this to prevent results from being biased in favour of one instrument over the other. This was due to our concern that an “order effect” might be induced [45], in which 5L-VAS responses were influenced by responding to the EQ-5D-5L directly beforehand and 3L-VAS responses were influenced by responding to the EQ-5D-3L directly beforehand.

Furthermore, as the sample size of proxy EQ-5D responses gathered was too small, and the participants from whom proxy responses were elicited had more physical health impairments than self-reporting participants, we removed proxy responders from our analyses. It was not feasible under these circumstances to investigate the measurement properties of proxy EQ-5D responses for older multimorbid adults. However, a separate analysis comparing the patient and proxy responses that we collected is planned for a future publication. Another limitation was that there may be country differences but that, given the sample size and the heterogeneity of the sample, these could be confirmed or assessed in detail. Larger studies would be required for this. Furthermore, our sample presented with a notably large number of multi-morbidities (mean of 11.5; median of 10 concurrent chronic conditions); hence, caution in generalising our results to older adults with fewer comorbidities should be exercised.

Another possible limitation is that we decided to use the German crosswalk method to calculate 5L scores instead of the German 5L value set. We did this because using the crosswalk method instead of a national value set is still a recognized standard in some major guidelines for calculating 5L scores for economic evaluations [2], indicating this is currently best practice. The implications of this decision on the results from our study are not known.

One potential area of future research is to compare test–retest reliability of the EQ-5D-3L and the EQ-5D-5L. Investigation of this property was beyond the scope of this study and few prior studies comparing the EQ-5D-3L and EQ-5D-5L have investigated this property, which relates to how strongly correlated repeated EQ-5D scores are [16, 46].

Both the EQ-5D-3L and EQ-5D-5L demonstrated satisfactory performance in this study, thus justifying their use as measures for HRQoL studies and cost-utility analyses of older people with multimorbidity. However, prominent guidelines recommend to use the EQ-5D-5L consistently across all diseases and populations [2], and the overall consensus of the literature comparing the measurement properties of the 3L and 5L in different patient populations, is that the 5L exhibits better measurement properties compared to the 3L [16]. Nevertheless, it also needs to be considered that the 3L may be considered slightly less burdensome to complete than the 5L due to having fewer response options. Also, when compared to the 5L the appropriate value sets for the 3L are currently available more widely.

We conclude that both the EQ-5D-3L and EQ-5D-5L exhibit a reasonably high level of performance for measuring the health of older adults with substantial multimorbidity and associated polypharmacy and who display the ability to self-complete the questionnaires.

## Data Availability

The data that support the findings of this study are available from CTU Bern, University of Bern but restrictions apply to the availability of these data, which were used under license for the current study, and so are not publicly available. Data are however available from the authors if reasonable request is provided and permission of CTU Bern, University of Bern is granted.

## Appendix

See Tables 7, 8, 9, 10, 11, 12, 13, 14, 15, 16, 17 and 18.

**Table 7:** Descriptive statistics: reported level of problem by dimensions in EQ-5D-3L

Dimension	6 months n (%)	%	12 months n (%)	%	*p* value*
Mobility					
No problems	114	50.8	112	50.0	0.74
Some problems	107	47.7	106	47.3	
Confined to bed	3	1.3	6	2.7	
ANY problem	110	49.0	112	50.0	
Self-care					
No problems	189	84.3	183	81.7	0.45
Some problems	26	11.6	31	13.8	
Unable to wash/dress	9	4.0	10	4.4	
ANY problem	35	15.6	41	18.2	
Usual activities					
No problems	163	72.7	152	67.8	0.21
Some problems	57	25.4	63	28.1	
Unable to perform	4	1.7	9	4.0	
ANY problem	61	27.1	72	32.1	
Pain/discomfort					
No pain/discomfort	113	50.4	122	54.4	0.38
Moderate pain/discomfort	97	43.3	90	40.1	
Extreme pain/discomfort	14	6.2	12	5.3	
ANY problem	111	49.5	102	45.4	
Anxiety/depression					
Not anxious or depressed	187	83.4	182	81.2	0.54
Moderately anxious/depressed	33	14.7	38	16.9	
Extremely anxious/depressed	4	1.7	4	1.7	
ANY problem	37	16.4	42	18.6	
No problem on all dimensions	66	29.4	66	29.4	

*Non-parametric test for trend across ordered groups developed by Cuzick [47]. We used this to test for differences between 6 and 12 months in responses for each EQ-5D-3L item

**At 12 months, 224 participants self-reported the EQ-5D-3L in full; 19 provided no response to 3L; 13 had 3L proxy reported by next of kin

**Table 8:** Descriptive statistics: reported level of problem by dimensions in EQ-5D-5L

Dimension	6 months n	%	12 months n	%	*p* value*
Mobility					
No problems	88	39.2	90	40.1	0.87
Slight problems	60	26.7	49	21.8	
Moderate problems	44	19.6	56	25.0	
Severe problems	27	12.0	24	10.7	
Unable to walk around	5	2.2	5	2.2	
ANY problem	136	60.5	134	59.7	
Self-care					
No problems	187	83.4	177	79.0	0.21
Slight problems	15	6.7	14	6.2	
Moderate problems	8	3.5	18	8.0	
Severe problems	7	3.1	6	2.6	
Unable to wash or dress	7	3.1	9	4.0	
ANY problem	37	16.5	47	21.0	
Usual activities					
No problems	143	63.8	136	60.7	0.36
Slight problems	35	15.6	32	14.2	
Moderate problems	34	15.1	40	17.8	
Severe problems	10	4.4	10	4.4	
Unable to do usual activities	2	0.8	6	2.6	
ANY problem	81	36.2	88	39.3	
Pain/discomfort					
No pain/discomfort	100	44.6	111	49.5	0.50
Slight pain/discomfort	46	20.5	37	16.5	
Moderate pain/discomfort	49	21.8	47	20.9	
Severe pain/discomfort	29	12.9	26	11.6	
Extreme pain/discomfort	0	0.0	3	1.3	
ANY problem	124	55.4	113	50.4	
Anxiety/depression					
Not anxious or depressed	177	79.0	178	79.4	0.92
Slightly anxious/depressed	28	12.5	26	11.6	
Moderately anxious/depressed	12	5.3	13	5.8	
Severely anxious/depressed	5	2.2	6	2.6	
Extremely anxious/depressed	2	0.8	1	0.4	
ANY problem	47	21.0	46	20.5	
No problem on all dimensions	50	22.3	49	21.9	

*Non-parametric test for trend across ordered groups developed by Cuzick [47]. We used this to test for differences between 6 and 12 months in responses for each EQ-5D-5L item

**At 12 months, 226 participants self-reported the EQ-5D-5L in full; 15 provided no response to 5L, 14 had 5L proxy reported by next of kin; 1 partially reported the 5L by responding only to the 5L usual activities, pain/discomfort and anxiety/depression items

**Table 9:** “Any” problem rates reported using 3L / 5L at 6 and 12 months (%)

	6 months 3L	12 months 3L	Change in rate (3L) %	6 months 5L	12 months 5L	Change in rate (5L) %
Mobility	49.1	50.0	0.9	60.7	59.8	− 0.9
Self-care	15.6	18.3	2.7	16.5	21.0	4.5
Usual activities	27.2	32.1	4.9	36.2	39.3	3.1
Pain/discomfort	49.6	45.5	− 4.0	55.4	50.4	− 4.9
Anxiety/depression	16.5	18.8	2.2	21.0	20.5	− 0.4

**Table 10:** Cross-tabulation of 3L and 5L responses at 6 months (n = 224)

3L	5L				
Mobility	No problems	Some problems	Moderate problems	Severe problems	Unable
No problems	85	25	**4**	0	0
Some problems	**3**	35	40	27	2
Confined to bed	0	0	0	0	3

3L	5L				
Self-care	No problems	Some problems	Moderate problems	Severe problems	Unable
No problems	184	5	0	0	0
Some problems	**3**	10	7	6	0
Unable	0	0	**1**	1	7

3L	5L				
Usual activities	No problems	Some problems	Moderate problems	Severe problems	Unable
No problems	142	15	**6**	0	0
Some problems	**1**	20	28	8	0
Unable	0	0	0	2	2

3L	5L				
Pain/discomfort	No problems	Some problems	Moderate problems	Severe problems	Extreme
None	98	13	**2**	0	0
Moderate	**2**	33	47	15	0
Extreme	0	0	0	14	0

3L	5L				
Anxiety/depression	No problems	Some problems	Moderate problems	Severe problems	Extreme
None	176	11	0	0	0
Moderate	**1**	17	11	2	**2**
Extreme	0	0	**1**	3	0

*Bold values represent inconsistent responses. An inconsistent response was defined as an EQ-5D-5L response which was two or more levels away from the respondent’s EQ-5D-3L response. An exception to this rule was made for the mobility item. Here, we considered responses from participants who reported with the 3L some problems in walking about, and also reported with the 5L being unable to walk about, to *not* be inconsistent. This is because the 3L mobility item is categorised into a person having “no problems in walking about”, “some problems in walking about” and being “confined to bed”. People who report being unable to walk about with the 5L, may not necessarily be confined to bed and may therefore instead logically report having “some problems in walking about” with the 3L

**Table 11:** Assessment of responsiveness of the EQ-5D-3L and the EQ-5D-5L measures to changes in the Barthel Index, for the subgroup in Switzerland

	EQ-5D-3L at 6 months (mean)	EQ-5D-3L at 12 months (mean)	Difference between 12 and 6 months EQ-5D-3L (95% CI)	Effect size (Cohen’s D)^a^	n	% reporting 3L score increase
Barthel Index						
Improved	0.41	0.79	0.38 (0.04 to 0.71)	1.63*	4	100
No change	0.93	0.89	− 0.03 (− 0.10 to 0.02)	− 0.23	39	15
Worsened	0.84	0.70	− 0.13 (− 0.30 to 0.02)	− 0.54	12	17

	EQ-5D-5L at 6 months (mean)	EQ-5D-5L at 12 months (mean)	Difference between 12 and 6 months EQ-5D-5L (95% CI)	Effect size (Cohen’s D)	n	% reporting 5L score increase
Barthel Index						
Improved	0.40	0.73	0.32 (0.01 to 0.63)	1.49	4	100
No change	0.91	0.90	− 0.01 (− 0.05 to 0.02)	− 0.11	39	15
Worsened	0.78	0.71	− 0.06 (− 0.20 to 0.07)	− 0.28	12	33

^a^Cohen’s D effect sizes of between 0.2 and 0.5 are considered small, 0.5 and 0.8 moderate and > 0.8 large

**p* < 0.05

**Table 12:** Assessment of responsiveness of the EQ-5D-3L and the EQ-5D-5L measures to changes in the Barthel Index, for the subgroup in Belgium

	EQ-5D-3L at 6 months (mean)	EQ-5D-3L at 12 months (mean)	Difference between 12 and 6 months EQ-5D-3L (95% CI)	Effect size (Cohen’s D)^a^	n	% reporting 3L score increase
Barthel Index						
Improved	0.54	0.67	0.12 (− 0.21 to 0.45)	0.34	5	40
No change	0.84	0.84	0.00 (− 0.04 to 0.03)	0.00	37	22
Worsened	0.80	0.67	− 0.13 (− 0.29 to 0.03)	− 0.65	15	20

	EQ-5D-5L at 6 months (mean)	EQ-5D-5L at 12 months (mean)	Difference between 12 and 6 months EQ-5D-5L (95% CI)	Effect size (Cohen’s D)	n	% reporting 5L score increase
Barthel Index						
Improved	0.63	0.64	0.01 (− 0.09 to 0.11)	0.07	5	20
No change	0.81	0.81	0.00 (− 0.03 to 0.03)	0.00	37	38
Worsened	0.75	0.63	− 0.11 (− 0.24 to 0.01)	− 0.67	15	33

^a^Cohen’s D effect sizes of between 0.2 and 0.5 are considered small, 0.5 and 0.8 moderate and > 0.8 large

**Table 13:** Assessment of responsiveness of the EQ-5D-3L and the EQ-5D-5L measures to changes in the Barthel Index, for the subgroup in Netherlands

	EQ-5D-3L at 6 months (mean)	EQ-5D-3L at 12 months (mean)	Difference between 12 and 6 months EQ-5D-3L (95% CI)	Effect size (Cohen’s D)^a^	n	% reporting 3L score increase
Barthel Index						
Improved	0.79	0.88	0.09 (0.00 to 0.18)	0.58	11	55
No change	0.87	0.90	0.02 (− 0.01 to 0.07)	0.22	37	41
Worsened	0.69	0.63	− 0.03 (− 0.13 to 0.07)	− 0.10	15	40

	EQ-5D-5L at 6 months (mean)	EQ-5D-5L at 12 months (mean)	Difference between 12 and 6 months EQ-5D-5L (95% CI)	Effect size (Cohen’s D)	n	% reporting 5L score increase
Barthel Index						
Improved	0.73	0.85	0.12 (0.02 to 0.22)	0.78	11	64
No change	0.84	0.87	0.03 (− 0.01 to 0.08)	0.21	37	43
Worsened	0.68	0.62	− 0.06 (− 0.15 to 0.03)	− 0.21	15	27

^a^Cohen’s D effect sizes of between 0.2 and 0.5 are considered small, 0.5 and 0.8 moderate and > 0.8 large

**Table 14:** Assessment of responsiveness of the EQ-5D-3L and the EQ-5D-5L measures to changes in the Barthel Index, for the subgroup in Ireland

	EQ-5D-3L at 6 months (mean)	EQ-5D-3L at 12 months (mean)	Difference between 12 and 6 months EQ-5D-3L (95% CI)	Effect size (Cohen’s D)^a^	n	% reporting 3L score increase
Barthel Index						
Improved	0.71	0.87	0.15 (− 0.05 to 0.36)	0.74	11	45
No change	0.90	0.84	− 0.05 (0.18 to 0.06)	− 0.25	29	24
Worsened	0.57	0.57	0.00 (− 0.29 to 0.29)	0.00	8	38

	EQ-5D-5L at 6 months (mean)	EQ-5D-5L at 12 months (mean)	Difference between 12 and 6 months EQ-5D-5L (95% CI)	Effect size (Cohen’s D)	n	% reporting 5L score increase
Barthel Index						
Improved	0.73	0.86	0.12 (− 0.01 to 0.26)	0.61	11	55
No change	0.89	0.83	− 0.05 (− 0.16 to 0.05)	− 0.25	29	31
Worsened	0.69	0.59	− 0.09 (− 0.30 to 0.10)	− 0.35	8	38

^a^Cohen’s D effect sizes of between 0.2 and 0.5 are considered small, 0.5 and 0.8 moderate and > 0.8 large

**Table 15:** Assessment of responsiveness of the EQ-5D-3L and the EQ-5D-5L measures to changes in the VAS, for the subgroup in Switzerland

	EQ-5D-3L at 6 months (mean)	EQ-5D-3L at 12 months (mean)	Difference between 12 and 6 months EQ-5D-3L (95% CI)	Effect size (Cohen’s D)^a^	n	% reporting 3L score increase
3L-VAS						
Improved	0.92	0.93	0.01 (− 0.03 to 0.05)	0.15	10	30
No change	0.87	0.81	− 0.06 (− 0.19 to 0.07)	− 0.22	25	24
Worsened	0.85	0.84	− 0.01 (− 0.08 to 0.06)	− 0.06	20	15

	EQ-5D-5L at 6 months (mean)	EQ-5D-5L at 12 months (mean)	Difference between 12 and 6 months EQ-5D-5L (95% CI)	Effect size (Cohen’s D)	n	% reporting 5L score increase
5L-VAS						
Improved	0.90	0.91	0.01 (− 0.02 to 0.04)	0.14	10	30
No change	0.83	0.81	− 0.02 (− 0.10 to 0.07)	− 0.06	25	28
Worsened	0.84	0.85	0.01 (− 0.06 to 0.08)	0.05	20	20

^a^Cohen’s D effect sizes of between 0.2 and 0.5 are considered small, 0.5 and 0.8 moderate and > 0.8 large

**Table 16:** Assessment of responsiveness of the EQ-5D-3L and the EQ-5D-5L measures to changes in the VAS, for the subgroup in Belgium

	EQ-5D-3L at 6 months (mean)	EQ-5D-3L at 12 months (mean)	Difference between 12 and 6 months EQ-5D-3L (95% CI)	Effect size (Cohen’s D)^a^	n	% reporting 3L score increase
3L-VAS						
Improved	0.82	0.84	0.02 (− 0.05 to 0.09)	0.25	6	33
No change	0.80	0.77	− 0.02 (− 0.11 to 0.06)	− 0.12	32	25
Worsened	0.80	0.77	− 0.02 (0.10 to 0.04)	− 0.13	20	20

	EQ-5D-5L at 6 months (mean)	EQ-5D-5L at 12 months (mean)	Difference between 12 and 6 months EQ-5D-5L (95% CI)	Effect size (Cohen’s D)	n	% reporting 5L score increase
5L-VAS						
Improved	0.70	0.70	0.00 (− 0.15 to 0.15)	− 0.01	9	33
No change	0.80	0.78	− 0.02 (− 0.07 to 0.01)	− 0.17	29	41
Worsened	0.78	0.74	− 0.03 (− 0.12 to 0.05)	− 0.18	20	30

^a^Cohen’s D effect sizes of between 0.2 and 0.5 are considered small, 0.5 and 0.8 moderate and > 0.8 large

**Table 17:** Assessment of responsiveness of the EQ-5D-3L and the EQ-5D-5L measures to changes in the VAS, for the subgroup in Netherlands

	EQ-5D-3L at 6 months (mean)	EQ-5D-3L at 12 months (mean)	Difference between 12 and 6 months EQ-5D-3L (95% CI)	Effect size (Cohen’s D)^a^	n	% reporting 3L score increase
3L-VAS						
Improved	0.80	0.90	0.10 (0.02 to 0.17)	0.81*	13	69
No change	0.85	0.87	0.01 (− 0.02 to 0.06)	0.12	32	41
Worsened	0.75	0.74	− 0.01 (− 0.12 to 0.09)	− 0.05	18	28

	EQ-5D-5L at 6 months (mean)	EQ-5D-5L at 12 months (mean)	Difference between 12 and 6 months EQ-5D-5L (95% CI)	Effect size (Cohen’s D)	n	% reporting 5L score increase
5L-VAS						
Improved	0.75	0.87	0.11 (0.02 to 0.20)	0.78	13	62
No change	0.82	0.85	0.03 (− 0.01 to 0.06)	0.16	32	38
Worsened	0.73	0.69	− 0.04 (− 0.14 to 0.06)	− 0.14	18	39

^a^Cohen’s D effect sizes of between 0.2 and 0.5 are considered small, 0.5 and 0.8 moderate and > 0.8 large. *p < 0.05

**Table 18:** Assessment of responsiveness of the EQ-5D-3L and the EQ-5D-5L measures to changes in the VAS, for the subgroup in Ireland

	EQ-5D-3L at 6 months (mean)	EQ-5D-3L at 12 months (mean)	Difference between 12 and 6 months EQ-5D-3L (95% CI)	Effect size (Cohen’s D)^a^	n	% reporting 3L score increase
3L-VAS						
Improved	0.73	0.84	0.10 (− 0.02 to 0.24)	0.41	23	39
No change	0.92	0.71	− 0.20 (− 0.48 to 0.06)	− 0.67	12	25
Worsened	0.82	0.82	0.00 (− 0.08 to 0.09)	0.00	13	23

	EQ-5D-5L at 6 months (mean)	EQ-5D-5L at 12 months (mean)	Difference between 12 and 6 months EQ-5D-5L (95% CI)	Effect size (Cohen’s D)	n	% reporting 5L score increase
5L-VAS						
Improved	0.76	0.83	0.06 (0.00 to 0.14)	0.30	23	48
No change	0.91	0.72	− 0.19 (− 0.47 to 0.09)	− 0.61	12	33
Worsened	0.84	0.81	− 0.02 (− 0.07 to 0.01)	− 0.18	13	23

^a^Cohen’s D effect sizes of between 0.2 and 0.5 are considered small, 0.5 and 0.8 moderate and > 0.8 large
